# P-1032. Vein Attempts & IV League Hazards: Rethinking Peripheral IV and Bacteremia Risk

**DOI:** 10.1093/ofid/ofaf695.1228

**Published:** 2026-01-11

**Authors:** Roopali Dahiya, Jennifer Fede, Jennifer Gutowski, Kelly Cunningham, Melissa Bonstein, Emil P Lesho

**Affiliations:** Rochester General Hospital, Rochester, NY; Rochester Regional Health, Rochester, New York; Rochester Regional Health, Rochester, New York; Rochester Regional Health, Rochester, New York; Rochester Regional Health, Rochester, New York; Rochester Regional Health, Rochester, New York

## Abstract

**Background:**

Peripheral intravenous catheters (PIV) are the most common invasive devices in hospitalized patients, yet data pertaining to serious complications from PIV are scarce. After noticing an apparent uptick in such complications, we sought to determine the frequency and impact of PIV-related bacteremia from 01/01/2023 to 04/15/2025 at a 528-bed teaching hospital in upstate New York.

**Table 1:**
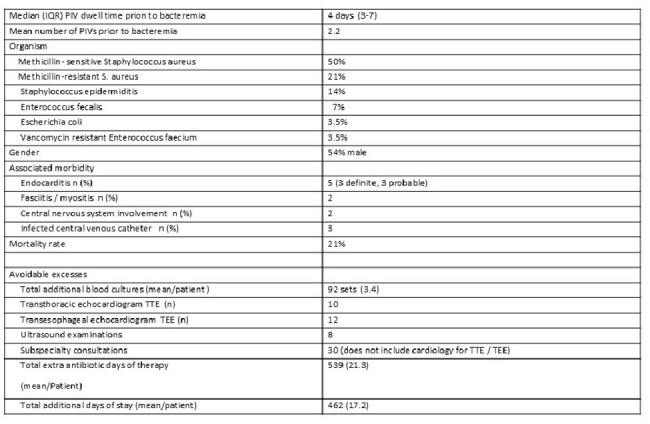
Characteristics of Peripheral Intravenous Catheter Associated Bacteremia (n = 28)

**Table 2:**
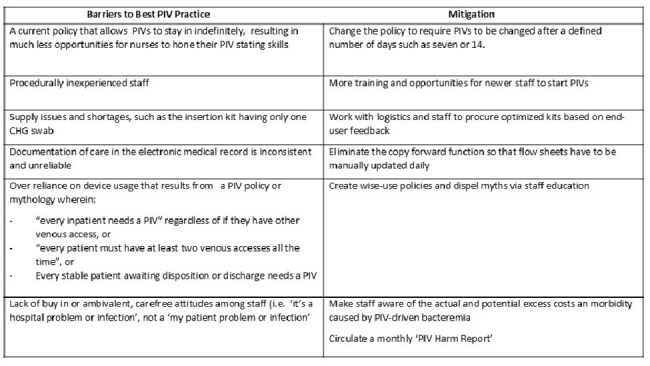
Barriers to Best Practices for Insertion and Maintenance of Peripheral Intravenous Catheters

**Methods:**

All blood cultures positive >/= 48 hrs. post admission were obtained via laboratory information system query. Number of PIV starts was obtained from census data. All patients with *Staphlococcus aureus* bacteremia (SaB) are routinely seen by infectious diseases providers who identify the source of all bacteremia. During mandatory reporting of hospital onset methicillin resistant SaB, the source of the bacteremia is also routinely identified. PIV-related adverse events were obtained from a virtual private network service (Safe Connect® McAfee) used to report serious safety-related issues, and via notifications from staff. Records of patients with proven PIV-driven bacteremia were retrospectively reviewed.

**Results:**

7477 PIV starts led to 28 bacteremias for a rate of 3.7 bacteremias/1,000 starts. 1534 positive blood cultures from 1017 unique patients were obtained. 2.8% (28/1017) of all hospital onset (HO) bacteremia had PIV as a definite source. Five species accounted for all PIV-driven bacteremia (PIV-B), 71% (n=20) were SaB (Table 1). 21% (6/28) of PIV-B were caused by MRSA. 25 HO-MRSA occurred. 24% 6/25 of HO MRSA had PIV as the source of the bacteremia. PIV-B was associated with the excess costs seen in Table 1, including a mean of 17.2 extra days of stay and 21.3 extra antibiotic days of therapy. PIV-B was also associated with endocarditis and central nervous system involvement. 21% of PIV-B were fatal.

**Conclusion:**

The morbidity, mortality and avoidable costs associated with PIV-B are substantial. Our findings highlight the criticality of using well-known best practices for insertion and maintenance. Barriers to those practices appear in Table 2.

**Disclosures:**

All Authors: No reported disclosures

